# Data on System Approach to Process of urban housing construction, renewal and upgrading

**DOI:** 10.1016/j.dib.2018.06.106

**Published:** 2018-07-03

**Authors:** Amusan Lekan, Osawaru Faith, Akanya Cinwosoko Ninma, Awotinde Oladipupo Sunday

**Affiliations:** Building Technology, College of Science and Technology, Covenant University, Nigeria

## Abstract

Data about system that could be used in urban housing Construction process, renewal and upgrading is presented in this data article. Urban upgrading has been widely recognized as an essential issue in developing sustainable built environment. The aim is to identify the system approach that could be used for renewing and upgrading urban housing generally with view to expanding cities, redevelopment, redesigning and beautification of settlement layout, upgrading of facilities and public goods and services, repair, construction and silting of drainage system. Stratified survey method was used in generating the data, through identifying the current housing system in some selected locations in Ota, Ogun State, Nigeria, examining the factors that affects urban housing renewal and upgrading, identifying and examine the system approaches for urban housing renewal and upgrading and to develop a template for alternative material intervention for urban housing. The data was generated through questionnaire survey of 100 respondents; through Stratified sampling technique. Data collected were analyzed using Statistical Package for Social Sciences (SPSS) with Descriptive Statistics such as percentage distribution, charts and relative agreement index for the interpretation of findings. Data was presented on system that could be used in urban construction process, renewal and upgrading as includes: Redevelopment system, Revitalization system, Rehabilitation system, Regeneration system, Integration system, Conservation system and Afforestation system.

**Specifications Table**

**Table:** 

**Subject area**	***Construction, Urban and Planning***
**More specific subject area**	*Housing*
**Type of data**	*Tables*
**How data was acquired**	*The Data was gathered through survey*
**Data format**	*Filtered analyzed*
**Experimental factors**	*Samples were carefully picked using stratified method.* Simple percentages and severity index were used as analytical tool for the generated data. SPSS (Statistical Packages for Social Science Students) was used in determining pattern of relationship among the cost determinants and variables. The factors were ranked in order of their degree of severity
**Experimental features**	*Questionnaire was used to collate data as the only source of data collection. Primary data were collected from the dwellers of the urban communities through qualitative and quantitative data using an interview guide, questionnaire and physical examination of the houses and its environment (snapshot). Population of the study consisted of 180 urban dwellers out of which a sample size of 90 was picked*
**Data source location**	Iyesi, Sango and Ota. Ogun State. Nigeria
**Data accessibility**	http://eprints.covenantuniversity.edu.ng/2194/#.WusYQO8vwdU
**Related research article**	*Urban Renewal and upgrading Systems*

**Value of the data**

• The data is useful in research that entails studying Urban Housing Renewal and Upgrading and the performance of construction projects [1], [2].
• Data presented is useful in studying Urban renewal system, urban upgrading and renewal/rehabilitation and their construction process [2].
• The data could be used in modeling of urban upgrading and renewal techniques [3].
• The data is valuable to construction project professionals and could be used in policy formulation [3].
• The data could be used as basis of comparison with that of other countries of the world in order to identify the uniqueness [4], [5], [6], [7], [8].

## 1 Data

The following data are presented in this Journal Article: Alternative Materials for urban housing renewal and upgrading, Data on Interventional Approach for urban housing renewal and upgrading; Data on Current Housing System by Usage; Data on Current Housing System by Material, Data on Factors that affect urban housing renewal and upgrading [7], [9].

## 2 Experimental design, materials, and methods

The Population of the study consisted of 180 urban houses [8], [9], [10]. A sample size of 90 was used for the analysis. Questionnaire was purposely distributed to respondents within the urban communities that were sampled for renewal and upgrading so as to air their views. Primary data were collected from the dwellers of the urban communities through qualitative and quantitative data using questionnaire and physical examination of the houses and its environment (snapshot).The data collected were analyzed using descriptive statistics. Descriptive Statistics such as percentage distribution and relative agreement index (ranking) for the analysis and interpretation of findings. The respondents were asked to indicate the level of agreement/disagreement using some selected methods on a 1–5 Likert-scale of Strongly agree (5), Agree (4), Strongly Disagree (3), Disagree (2) and Neutral (1).

Table 1 shows the percentage of age of the respondents, 35.78% were between the age group of 26–35 years, 31.11% of the respondents were in 36–50 years age group, 17.78% of the respondents from 18–25 years while, 13.33% of the respondents were between the ages of 50years and above respectively.

**Table 1:** Data on percentage of age of respondents.

Age of Respondents	Frequency	Percentage (%)
18–25 years	16	17.78
26–35 years	34	35.78
36–50 years	28	31.11
50 years and Above	12	13.33
Total	90	100%

Table 2 present the percentage distribution of respondents by sex shows that the bulk of the respondents were male with 51.11% and female with 48.89%. This result indicates that the locations sampled are populated with male gender.

**Table 2:** Data on percentage of gender of the respondents.

Sex	Frequency	Percentage (%)
Male	46	51.11
Female	44	48.89
Total	90	100%

Table 3 above shows the assessment of the percentage of nationality, 100% of respondents are Nigerians. This is an indication that Nigerians are populated in the locations sampled.

**Table 3:** Data on percentage of nationality of the respondents.

Nationality	Frequency	Percentage (%)
Nigerian	90	100
Non-Nigerian	0	0
Total	90	100%

Table 4 reveals the employment status of the respondents. While, considering the responses in the table, it shows that majority of the respondents (42.22%) were employed, 40% were self-employed while 17.78% of the respondents were un-employed.

**Table 4:** Data on result of employment status of the respondents.

Employment Status	Frequency	Percentage (%)
Employed	60	42.22
Un-employed	16	17.78
Self-employed	18	40
Total	90	100%

The Table 5 above shows the percentage distribution of respondents Educational level which indicates that 44.44% of the respondents have HND/First degree as the highest percentage, followed by 24.44% as OND holders, 8.89% of the respondents have no formal education, 6.67% of respondents have Master/Higher degrees and GradeII/NCE qualification, while, 4.44% respondents have WAEC/NECO and Primary school holder. This is an indication that first degree is the respondent׳s highest educational qualification.

**Table 5:** Data on result of educational qualification of respondents.

Educational Qualification	Frequency	Percentage (%)
No formal Education	8	8.89
Primary school holder	4	4.44
WAEC/NECO	4	4.44
Grade II/ NCE	6	6.67
OND	22	24.44
First degree/HND	40	44.44
Masters/Higher degree	12	6.67
Total	90	100%

These results in Table 6 above, reveals the purpose for which each respondents housing are used. As majority of the respondents with 0.86 relative agreement index indicated that their houses are being used for residential purpose, followed by Commercial housing with 0.54 relative agreement index in which the respondents use their buildings for commercial purpose. And this is closely followed by Educational purpose with 0.51 index and Agricultural Housing purpose with 0.49 index. Also, respondents housing usage shows that Recreational and Financial are on the same index with 0.47. The least housing system used by respondents is the Social Housing with 0.44 index. This result implies that the housing system within the area sampled are mostly for residential use. The data presented can be useful to planners in planning for the development of more residential houses and also it can help researchers in knowing the type of housing that is most important within a study area when carrying out a research [11].

**Table 6:** Data on current housing system by usage.

**S/n**	**Usage (type)**	**S.A**	**A**	**N**	**D**	**S.D**	**R.A.I**	**RANK**
**1**	Residential Housing	48	30	0	12	0	0.858	1
**2**	Educational Housing	8	8	8	36	24	0.514	3
**3**	Cultural Housing	0	0	28	42	28	0.433	7
**4**	Financial Housing	0	6	8	42	28	0.476	5
**5**	Industrial Housing	0	4	10	38	32	0.471	6
**6**	Legal Housing	2	2	10	40	30	0.471	6
**7**	Social Housing	0	0	14	38	32	0.443	7
**8**	Commercial Housing	6	10	8	16	28	0.538	2
**9**	Recreational Housing	2	2	10	38	32	0.476	5
**10**	Agricultural Housing	4	2	8	38	32	0.495	4

**Legend**: S.A=Strongly Agree(5) A=Agree(4) N=Neutral(1) S.D=Strongly Disagree(3) D=Disagree(2).

Table 7 shows the materials in which the respondents’ houses are made of. Sandcrete block housing shows the highest ranked materials used by respondents with 0.86 relative agreement index. Brick housing shows the second highest ranked with 0.58, followed by Iron housing with 0.48. Also, Fibre and wood housing have almost equal relative agreement index of 0.46 and the least appear to be Mud housing by material with 0.44 relative agreement index. This indicates that sandcrete material is mostly used and Mud material is least used in the area sampled. The data can help the planners to know the current trend on the use of materials for housing. Also, sandcrete and brick are most available for construction work. (Table 8).

**Table 7:** Data on current housing system by material.

**S/n**	**Materials (type)**	**S.A**	**A**	**N**	**D**	**S.D**	**R.A.I**	**RANK**
**1**	Mud Housing	0	0	12	46	32	0.444	6
**2**	Sandcrete Housing	23	36	4	6	0	0.862	1
**3**	Wood Housing	2	0	20	24	15	0.458	5
**4**	Iron Housing	0	0	20	17	23	0.480	3
**5**	Fibre Housing	0	0	12	38	20	0.462	4
**6**	Brick housing	2	10	8	19	16	0.511	2

**Legend**: S.A=Strongly Agree(5) A=Agree(4) N=Neutral(1) S.D=Strongly Disagree(3) D=Disagree(2).

**Table 8:** Data on factors that affect urban housing renewal and upgrading.

S/n	Factors	R.A.I	RANK
A	**PLANNING FACTORS**		
**1**	Dilapidated buildings	0.596	6
**2**	Inaccessibility	0.867	3
**3**	Poor physical planning	0.862	4
**4**	Waste management	0.893	1
**5**	Flooding	0.893	1
**6**	Lack of management regulations	0.596	6
**7**	Insufficient living space	0.680	5
**8**	Lack of access to electricity	0.587	8
**9**	Nature of decision making	0.342	10
**10**	Style of leadership	0.40	9
B	**PHYSICAL FACTOR**	**R.A.I**	**RANK**
**1**	Abandoned buildings	0.440	8
**2**	Slum	0.382	10
**3**	Improper maintenance	0.809	5
**4**	Poor ventilation	0.818	4
**5**	Inadequate housing	0.809	5
**6**	Lack of services and facilities	0.902	1
**7**	Narrow streets	0.898	2
**8**	Difficult access	0.844	3
**9**	Underused/vacant lands	0.418	9
**10**	Overcrowding	0.640	7
C	**SOCIO-CULTURAL FACTORS**		
**1**	Traditional and neighbourhood housing setting	0.533	2
**2**	Religious/Ethnic reasons	0.396	4
**3**	Loss of family land	0.431	3
**4**	Crime/Insecurity	0.809	1
**5**	Presence of family grave	0.364	5
D	**ECONOMIC FACTORS**		
**1**	Poverty	0.871	3
**2**	Loss of means of livelihood	0.876	2
**3**	Financial strain/Low income	0.938	1
**4**	Population	0.813	5
**5**	Unemployment	0.862	4
**6**	Lack of skills	0.684	6
E	**SOCIAL FACTOR**		
**1**	Poor health	0.449	6
**2**	Social deprivation	0.627	2
**3**	Large family size	0.573	4
**4**	Lack of education	0.618	3
**5**	Lack of social services	0.796	1
**6**	Lack of right to occupancy	0.422	7
**7**	Lack of recreational area	0.520	5
F	**ENVIRONMENTAL FACTOR**		
**1**	Negative behaviour of individual	0.329	8
**2**	High population	0.702	5
**3**	Absence of water	0.551	7
**4**	Absence of sanitation	0.884	4
**5**	Spread of disease	0.307	9
**6**	Lack of services	0.858	3
**7**	Lack of access to transportation and communication	0.516	6
**8**	Ignorance	0.276	10
**9**	Shortage of infrastructure	0.889	2
**10**	Poor disposal system	0.902	1

**Legend**: S.A=Strongly Agree(5) A=Agree(4) N=Neutral(1) S.D=Strongly Disagree(3) D=Disagree.

### 2.1 Planning factor

The result above shows the planning factors affecting the urban housing construction, renewal and upgrading in the areas sampled. As majority of the respondents are been affected by these factors. Flooding and waste management ranked highest with 0.89 relative agreement index. This is closely followed by inaccessibility and poor physical planning with 0.86 relative agreement index, insufficient living space has 0.68, also dilapidated buildings, lack of management regulation and lack of electricity with 0.59 relative agreement index respectively. The least factor which is Nature of decision making was ranked 0.34 respectively [12].

### 2.2 Physical factor

The physical factors that affect urban housing construction, renewal and upgrading in the area sampled. Data from the table above reveals that lack of services and facilities ranked the highest with 0.90 relative agreement index, followed by narrow streets with 0.89 index and difficult access with 0.84. Also, inadequate housing and improper maintenance were the same index as 0.81. While, abandoned buildings and vacant lands were ranked closely the same as 0.44 and 0.42. Slum was ranked least with 0.67 relative agreement index. Apart from these factors affecting urban housing renewal and upgrading, it also means that it can affect residents physiologically within their environment.

### 2.3 Socio-cultural factor

From the result, the highest ranked socio-cultural factor that affects urban renewal and upgrading within the area sampled is insecurity with 0.81 relative agreement index. Followed by traditional and neighbourhood setting as 0.53 relative agreement index, Also, loss of family land has 0.43 index. Finally, religious reasons and presence of family grave happen to be the least with 0.39 and 0.36 relative agreement index. This means that renewal and upgrading of the area can be threatened by insecurity having the highest rank.

### 2.4 Economic factor

Financial strain/low income is the highest ranked economic factors that affect occupants in the area sampled with 0.94 relative agreement index. Loss of means of livelihood and poverty with 0.87 index. Others include population and unemployment with 0.86 and 0.81 index. While, lack of skills has the least with 0.68 relative agreement index respectively.

### 2.5 Social factor

The result reveals that lack of social services rank the highest with 0.79 relative agreement index of the social factors that affect urban housing renewal and upgrading. From the same result obtained, social deprivation follows with 0.63, while Lack of education and large family size have 0.62 and 0.57 relative agreement index and finally, poor health is ranked the least with 0.45 relative agreement index.

### 2.6 Environmental factor

The Table 8 also shows the environmental factors that affect urban housing renewal and upgrading in the areas sampled. Based on the result as shown, it was discovered that poor disposal system was ranked the highest as 0.90 relative agreement index, shortage of infrastructure was ranked next as 0.89 relative agreement index and closely followed by absence of sanitation with 0.88. These factors are followed by lack of services with 0.86. High population has 0.70 relative agreement index. Also, negative behavior of individual and spread of disease have 0.32 and 0.31 index respectively, while ignorance happen to be the lowest index as 0.27.

The data in the table above could help builders, architects, engineers and other construction professionals on the factors to be taken into consideration in urban housing renewal and upgrading.

Table 9 shows the results obtained from respondents, the highest ranked preferred system approach for urban housing renewal and upgrading is Rehabilitation system with 0.91 relative agreement index. Which is followed by Redevelopment system with 0.87 index. And Regeneration and Conservation are closely ranked having 0.78 and 0.72 respectively. While, the least relative agreement index system approach are Integration, Revitalization and Afforestation. This is an indication that most respondents prefer their areas to be rehabilitated, redeveloped, or regenerated in the first three ranking, depending on their needs. The data shows the type of interventional system approach that urban developers and planners can use. It provides the steps to follow in urban planning and upgrading[13] (Table 10).

**Table 9:** Data on interventional system approach for urban housing construction, renewal and upgrading.

S/n	Interventional Approach	S.A	A	N	D	S.D	R.A.I	RANK
**1**	Re-development system	58	24	8	0	0	0.876	2
**2**	Rehabilitation system	54	34	0	2	0	0.911	1
**3**	Integration System	4	8	28	46	4	0.409	5
**4**	Revitalization system	2	22	64	2	0	0.369	6
**5**	Regeneration System	14	68	8	0	0	0.778	3
**6**	Conservation system	18	50	12	10	0	0.716	4
**7**	Afforestation system	0	10	52	24	4	0.338	7

**Table 10:** Rotation factor for system approach for rural-urban renewal and upgrade.

**S/N**	**FACTORS**	**F1**	**F2**	**F3**	**F4**	**F5**	**F6**	**F7**	**F8**	**F9**
1	Identification of areas that need redevelopment	0.339							0.392	1
2	Identifying opportunities involved in implementing the redevelopment programme							0.387	1	
3	Study the immediate reasons for inducing the redevelopment of the area					0.375		1		
4	Formulating an adequate redevelopment policy						1			
5	Identification of drivers of barriers to an effective redevelopment process					1				
6	Identification of problem in the area for rehabilitation				1					
7	Feasibility study for rehabilitation process			1						
8	Financing and setting up process		1							
9	Planning and implementing rehabilitation programme	1								
		**F10**	**F11**	**F12**	**F13**	**F14**	**F15**	**F16**	**F17**	**F18**
10	Identification of the state of deforestation of the landscape	1	0.387							
11	Feasibility study of topographical landscape		1							
12	Possibility of importing wood, trees and shrub species			1	0.388					
13	Identification of erosion impact in the landscape				1		0.391			
14	Charging of the soil with humors soil					1		0.383		
15	Culturing or planting of trees and shrub species on the area that needs afforestation						1			0.395
16	Identification of problem for integration							1		
17	Feasibility study of environment to be integrated								1	
18	Financing and setting up plans for integration									1
		**F19**	**F20**	**F21**	**F22**	**F23**	**F24**	**F25**	**F26**	**F27**
19	Implementation of the integration plans								0.398	1
20	Defining the need for regeneration in the environment						0.392		1	
21	Scoping of the environment							1		
22	Planning process for the regeneration					0.387	1			
23	Financing the regeneration programme		0.350		0.396	1				
24	Implementing the regeneration plan				1					
25	Investigation about the need for neighbourhood revitalization			1						
26	Identification of portion in the area that needs to be revitalized		1							
27	Provision of amenities to abandoned and deplorable communities	1								
		**F28**	**F29**	**F30**	**F31**	**F32**	**F33**	**F34**	**F35**	**F36**
28	Introduction of revitalization agent such as: pipe-borne water, electricity etc.	1	0.377							
29	Setting up of system to ensure continual flow of resources into the environment		1	0.388						
30	Identify nature of conservation system that is required in the particular location			1		0.389				
31	Carrying out the comprehensive analysis of the features that needs to be protected				1			0.392		
32	SWOT analysis of implementing strategy involved in environmental conservation					1			0.413	
33	Identification of challenges that could be encountered in the conservation policy						1			
34	Formulating strategy for monitoring and feedback on the conserved environment							1		0.407

Factor rotation operation was carried out on the system approach parameters using SPSS software. Factors with Eigen values that spans within 0 and 1 was adopted in selecting parameters for the above details presented in Table 10. This rotation method was used to generate the parameters to a sizeable number. However, some items were removed since their factor loadings didn’t fall between 0 and 1. Each of the factors remaining was grouped under Nine (9) models as listed below:

Model 1=0.339F_1_
Model 2=0.387F_11_+0.350F_20_+0.377F_29_
Model 3=0.388F_30_
Model 4=0.388F_13_+0.396F_22_
Model 5=0.375F_5_+0.387F_23_+0.389F_32_
Model 6=0.391F_15_+0.392F_24_
Model 7=0.387F_7_+0.383F_16_+0.392F_34_
Model 8=0.392F_8_+0.398F_26_+0.413F_35_
Model 9=0.395F_18_+0.402F_36_
Model 1=0.339F_1_
Model 2=0.338F_30_
Model 3=0.387F_11_+0.350F_20_
Model 4=0.388F_13_+0.396F_22_
Model 5=0.391F_15_+0.392F_24_
Model 6=0.402F_36_+0.395F_18_
Model 7=0.375F_5_+0.387F_23_+0.389F_32_
Model 8=0.387F_7_+0.383F_16_+0.392F_34_
Model 9=0.392F_8_+0.398F_26_+0.413F_35_

### 2.7 Model Content Interpretation

Model 1=(0.339F_1_ Redevelopment System)
Model 2=(0.338F_30_ System)
Model 3=(0.387F_11_ Afforestation+0.350F_20_ Regeneration)
Model 4=(0.388F_13_ Afforestation+0.396F_22_Regeneration)
Model 5=(0.391F_15_ Afforestation+0.392F_24_Regeneration)
Model 6=(0.402F_36_ Conservation+0.395F_18_Afforestation)
Model 7=(0.375F_5_ Redevelopment+0.387F_23_ Regeneration+0.389F_32_Conservation)
Model 8=(0.387F_7_ Redevelopment+0.383F_16_ Afforestation+0.392F_34_Conservation)
Model 9=(0.392F_8_ Redevelopment+0.398F_26_ Integration+0.413F_35_Conservation)

**LEGEND**

F_1_=Identification of areas that need redevelopment.

F_30_=Setting up of revitalization system to ensure continual flow of resources into the environment.

F_11_=Identification of the state of deforestation of the landscape.

F_20_=Financing the regeneration programme.

F_13_=Possibility of importing wood, trees and shrub species.

F_22_=Financing the regeneration programme

F_15_=Identification of erosion impact in the landscape.

F_24_=Defining the need for regeneration in the environment.

F_36_=Introduction of revitalization agent such as: pipe-borne water, electricity etc.

F_18_=Culturing or planting of trees and shrub species on the area that needs afforestation.

F_5_=Study the immediate reasons for inducing the redevelopment of the area

F_23_=Planning process for the regeneration.

F_32_=Identify nature of conservation system that is required in the particular location.

F_7_=Identifying opportunities involved in implementing the redevelopment programme.

F_16_=Charging of the soil with humors soil.

F_34_=Carrying out the comprehensive analysis of the features that needs to be protected

F_8_=Identification of areas that need redevelopment.

F_26_=Implementation of the integration plans.

F_35_=SWOT analysis of implementing strategy involved in environmental conservation.

The purpose of this correlation matrix is to analyze the factors to be considered when adopting rural-urban renewal and upgrading system approach in environmental development. The factors helps to model the parameters of the component for environmental development system and to rank their level of importance and also to distinguish their relationship with one another. This result above is an indication that the nine models can be used separately or jointly depending on area of need in Rural and Urban upgrading and development.

The data presented in the nine models can be used in combination or alternatively depending on the problem at hand. This can serve as a watershed to further research in Technology of System that could be adopted in Urban/Rural Upgrading and development research. The Hedonic models has presented in multivariate nature a Pareto optimal alternatives in the form of nine models that could further be expanded through research to provide an integrated approach to selecting an optimal alternatives in environmental problems that cut across Environmental redevelopment, Environmental Revitalization, Afforestation, Regeneration, Environmental Conservation and Environmental Integration.

From Table 11 above, the highest top three ranked responses for the most preferred alternative materials to be used for renewing and upgrading the areas sample are Aluminum, paint and marble with 0.92 relative agreement index, which is closely followed by Sandcrete block with 0.91 relative agreement index. While, fiber cladding and terrazzo and stabilized laterite earth brick has 0.34 relative agreement index which fall in the least ranked category of materials to be used

**Table 11:** Alternative materials for urban housing renewal and upgrading.

S/N	Alternative building materials	S.A	A	N	D	S.D	R.A.I	RANK
**1**	Asbestos sheet	4	14	52	20	0	0.373	20
**2**	Aluminium sheet	56	34	0	0	0	0.924	1
**3**	Terrazzo	0	10	46	34	0	0.342	25
**4**	Cement floor and wall tiles	18	30	38	4	0	0.569	6
**5**	Paints	52	38	0	0	0	0.916	2
**6**	Stabilized laterite earth brick	4	4	46	34	2	0.347	24
**7**	Bamboo	0	0	10	38	42	0.471	8
**8**	Stones and rock	0	0	14	38	37	0.453	11
**9**	Plywood	0	2	12	42	34	0.458	9
**10**	Sandcrete blocks	56	32	2	0	0	0.911	4
**11**	Burnt bricks	0	4	14	64	16	0.404	15
**12**	Steel reinforcement	4	18	62	8	0	0.356	23
**13**	Timber	0	12	22	48	8	0.422	14
**14**	Fibre cladding	0	6	48	34	2	0.342	25
**15**	Polythene	0	0	20	64	6	0.369	21
**16**	Glass	20	66	4	0	0	0.640	5
**17**	Laminated polyester	0	16	34	44	8	0.360	22
**18**	Hydro foam	4	12	30	32	8	0.458	9
**19**	Particle board	4	10	40	36	0	0.382	17
**20**	Asphalt	4	34	26	30	0	0.516	7
**21**	Limestone	4	2	28	50	6	0.387	16
**22**	Gypsum	0	32	52	6	0	0.427	13
**23**	Marble	31	24	2	2	0	0.916	2
**24**	Clay	0	0	6	58	26	0.444	12
**25**	Granite	9	49	46	12	2	0.382	17
**26**	Fibre board	0	4	30	42	14	0.382	17

**Legend**: S.A=Strongly Agree(5) A=Agree(4) N=Neutral(1) S.D=Strongly Disagree(3) D=Disagree(2).

The data provides various alternative building materials that can be used when applying the interventional system approaches in urban renewal and upgrading. It consist of both local and foreign materials [14], [15].
